# Trends in the Incidence of Central Precocious Puberty and Normal Variant Puberty Among Children in Denmark, 1998 to 2017

**DOI:** 10.1001/jamanetworkopen.2020.15665

**Published:** 2020-10-12

**Authors:** Elvira V. Bräuner, Alexander S. Busch, Camilla Eckert-Lind, Trine Koch, Martha Hickey, Anders Juul

**Affiliations:** 1 Department of Growth and Reproduction, Rigshospitalet, University of Copenhagen, Copenhagen, Denmark; 2 The International Research and Research Training Centre in Endocrine Disruption of Male Reproduction and Child Health (EDMaRC), Rigshospitalet, University of Copenhagen, Copenhagen, Denmark; 3 Department of Obstetrics and Gynaecology, University of Melbourne, Melbourne, Victoria, Australia

## Abstract

**Importance:**

There has been a worldwide secular trend toward earlier onset of puberty in the general population. However, it remains uncertain if these changes are paralleled with increased incidence of central precocious puberty (CPP) and normal variant puberty (ie, premature thelarche [PT] and premature adrenarche [PA]) because epidemiological evidence on the time trends in the incidence of these puberty disorders is scarce.

**Objective:**

To provide valid epidemiological data on the 20-year secular trend in the incidence rates of CPP and normal variant puberty.

**Design, Setting, and Participants:**

This population-based, 20-year cohort study used national registry data for all youth in Denmark registered with an incident diagnosis of CPP, PT, or PA in the Danish National Patient Registry from 1998 to 2017 (N = 8596) using the *International Statistical Classification of Diseases and Related Health Problems, Tenth Revision *(*ICD*-*10*). We applied the maximum diagnostic age limit for precocious puberty (ie, onset of puberty before age 8 years for girls and age 9 years for boys) with and without a 12-month lag to address time from first contact to final registration in the Danish National Patient Registry. Data analysis was conducted in 2019.

**Exposures:**

Diagnosis of CPP, PT, or PA.

**Main Outcomes and Measures:**

The age-specific and sex-specific incidence rates of first-time diagnosis of CPP, PT, and PA were estimated using data from the Danish National Patient Registry from 1998 to 2017, and information about the total number of children at risk within the same age groups and sex from Statistics Denmark. Incidences were stratified according to immigration group (Danish origin, first-generation immigrant, second-generation immigrant).

**Results:**

Overall a total 8596 children (7770 [90.4%] girls; median [interquartile] age at diagnosis for boys, 8.0 [7.1-9.0] years; for girls, 8.0 [7.6-8.5] years) were registered with an incident diagnosis of CPP, PT, or PA, of whom 7391 (86.0%) had Danish origin (6671 [90.3%] girls), corresponding to 370 new cases in children with Danish origin per year. The 20-year mean annual incidence rates of CPP, PT, PA, and all 3 conditions per 10 000 girls with Danish origin were 9.2 (95% CI, 8.0 to 10.3), 1.1 (95% CI, 0.7 to 1.5), 1.3 (95% CI, 0.9 to 1.7), and 11.5 (95% CI, 10.3 to 12.8), respectively. For boys with Danish origin, the 20-year mean annual incidence rates per 10 000 boys were lower: 0.9 (95% CI, 0.6 to 1.2), 0.2 (95% CI, 0.1 to 0.4), and 1.1 (95% CI, 0.7 to 1.4) for CPP, PA, and the sum, respectively. There was a 6-fold increase in incidence for girls with Danish origin (from 2.6 per 10 000 to 14.6 per 10 000) and a 15-fold increase for boys with Danish origin (from 0.1 per 10 000 to 2.1 per 10 000). The 20-year mean incidence of CPP and PA among girls in the first-generation and second-generation immigrant groups were greater than that of girls with Danish origin. The incidence rate for CPP per 10 000 girls in the first-generation and second-generation groups were 13.7 (95% CI, 9.3 to 18.2) and 14.2 (95% CI, 4.6 to 23.9), respectively; the incidence rate for PA per 10 000 girls in the first-generation and second-generation groups were 2.0 (95% CI, 0.3 to 3.6) and 1.5 (95% CI, −1.6 to 4.7), respectively. No differences associated with immigration status were observed among boys.

**Conclusions and Relevance:**

Our findings suggest that the annual incidence of CPP and normal variant puberty has substantially increased in Denmark during the last 20 years. These findings have implications for short-term and long-term health and potentially for the international classification of the reference age of puberty.

## Key Points

**Question:**

Have the annual incidence rates of central precocious puberty (CPP) and normal variant puberty (ie, premature thelarche and premature adrenarche) increased during the past 20 years in Denmark?

**Findings:**

This cohort study of 8596 children in Denmark who were diagnosed with CPP, premature thelarche, or premature adrenarche between 1998 and 2017 found that the annual incidence of CPP and normal variant puberty has increased substantially in Denmark during the last 20 years.

**Meaning:**

The findings of this study have implications for short-term and long-term health and potentially for the international classification of the reference age at puberty.

## Teaser

This cohort study provides epidemiological data on the 20-year secular trend in the incidence rates of central precocious puberty and normal variant puberty among children in Denmark.

## Introduction

Puberty marks the dynamic transition from childhood to the mature adult phenotype with full reproductive capacity. In girls, puberty most commonly starts with breast development (thelarche) and is followed by the first menstrual bleeding (menarche) approximately 3 years later. Timing of puberty exhibits wide interindividual variation (age 8-13 years in girls; age 9-14 years in boys), and known factors regulating timing and tempo of puberty include familial history and genetics, lifestyle, nutrition, and environmental exposures.^1^

Understanding changes in the timing of puberty as well as increasing referrals of children with suspected premature puberty is important because earlier age at puberty may be associated with psychosocial difficulties, is a risk factor for younger age at first sexual intercourse, and carries negative implications for long-term health, including increased risk of type 2 diabetes,^2,3^ greater adiposity, weight gain, and obesity,^4,5^ cardiovascular disease,^2^ depression,^6^ and premature death.^7^ Large-scale genetic data suggest that early puberty is associated with increased risk of breast cancer,^8^ consistent with extensive epidemiological evidence that early menarche increases the risk of breast cancer. For men, early puberty may increase the risk of prostate cancer.^9^

During recent decades, there have been global reports of a secular trend toward earlier onset of puberty in the general population, first reported among girls in the United States in the 1990s^10,11,12,13^ and subsequently in boys.^13,14^ European data from the same period did not replicate these findings,^15,16^ but 20 years later, European data (in 2005-2006) suggested a similar trend toward earlier onset of puberty in girls^17,18^ and, to a lesser extent, in boys.^19^

This trend toward earlier onset of puberty in the general population has been paralleled by an increase in the number of children (mainly girls) referred for evaluation of suspected precocious puberty (PP).^20^ Precocious puberty is defined as the development of secondary sexual characteristics before age 8 years for girls and 9 years for boys^21^ and is more commonly diagnosed in girls than boys.^22^ In girls, central (or true) precocious puberty (CPP) is generally idiopathic without lesions of the central nervous system, whereas boys are more likely to have an underlying pathological etiology.^23^ Classic CPP is defined by premature development of secondary sex characteristics, acceleration of linear growth, advanced bone age, and a pubertal response to a gonadotropin-releasing hormone (GnRH) test. By contrast, premature thelarche (PT) is defined as a self-limiting, isolated breast development associated with a normal growth rate, bone age corresponding to chronological age, and prepubertal response to a stimulation GnRH test. There are 2 forms of PT. The most common form occurs in infancy following the marked sex steroid production in minipuberty and the other at approximately age 5 to 8 years in girls. Premature adrenarche (PA) is a condition characterized by premature pubic hair development (pubarche), normal growth rate, and mild elevations of adrenal androgens after exclusion of serious adrenal disorders (ie, congenital adrenal hyperplasia or adrenal carcinoma).^22^ Both PT and PA are benign forms of PP and are associated with normal height potential and age at menarche.

To date, very few epidemiological studies of which we are aware have reported the national incidence of PP (only 5 previous studies^22,24,25,26,27^), and no studies to our knowledge have specifically reported incidences of PT and PA (Table 1). Our previous national Danish study^22^ found no secular upward trend in the annual incidence of PP during the 9-year period from 1993 to 2001. However, more recent European^24,25^ and Korean^26,27^ studies based on number of prescriptions, medication reimbursements, or data from health insurance agencies have suggested a possible upward trend in the incidence of CPP. Furthermore, a 2020 meta-analysis^28^ reported substantial worldwide changes in age at breast development among healthy girls. Early breast development is associated with adiposity and may not constitute full activation of the hypothalamo-pituitary-gonadal hormone axis, which results in progressive central puberty. Thus, age at menarche is also showing a trend toward earlier age of onset but less substantial than that observed for earlier thelarche. Thus, we do not know if the observed worldwide trend in earlier age at thelarche is associated with an increase in girls being evaluated, diagnosed with, and treated for CPP. Therefore, we set out to establish valid epidemiological data on the 20-year nationwide incidence of CPP and normal variant puberty, including PT and PA, in Denmark.

**Table 1.** Previous Studies Reporting National Incidence in Precocious Puberty

Source; country	Period	Data source	Diagnosis lag-time^a^	Disorder	Mean incidence per 10 000	
					Girls	Boys
Present study; Denmark ^b^	1998-2017	National patient registry	Yes, 1 y	CPP	9.2	0.9
				PT	1.1	NA
				PA	1.3	0.2
			No	CPP	7.7	0.8
				PT	1.2	NA
				PA	1.3	0.2
Teilman et al,^22^ 2005; Denmark	1993-2001	National patient registry	Yes, 1 y	CPP	3.0	0.4
Le Moal et al,^25^ 2018; France^c^	2011-2013	National insurance claims data	Yes, 1 y	CPP	2.6	0.24
Soriano-Guillen et al,^24^ 2010; Spain	2008-2009	Tertiary care centers	No	CPP	0.217	0.023
Kim et al,^26^ 2015; Korea	2004-2010	Korean Health Insurance Review Agency data	No	CPP	1.53	0.06
Kim et al,^27^ 2019; Korea	2008-2014	National insurance claims data	No	CPP	26.28	0.7

Abbreviations: CPP, central precocious puberty; NA, not applicable; PA, premature adrenarche; PT, premature thelarche.

^a^ The generally accepted maximum diagnostic age limit for precocious puberty remains 8 years for girls and 9 years for boys. There is an established 6-month to 12-month lag time between the first observation of signs of puberty reported by parents and the establishment of a record (ie, diagnosis by a pediatrician); this lag was included in 3 of 6 studies (50.0%).

^b^ Data in the present study were analyzed in main analyses with the 1-year lag for diagnosis (ie, at-risk girls aged 0-9 years; at-risk boys aged 0-10 years) and without the 1-year lag for diagnosis (ie, at-risk girls aged 0-8 years; at-risk boys aged 0-9 years).

^c^ Incidence was dependent on geographical location (proposed exposure to endocrine-disrupting agricultural chemicals) and varied several folds, ie, for girls from 0.96 to 12.39 per 10 000 and for boys 0.12 to 1.72 per 10 000.

## Methods

Research was conducted in accordance with principles of the Declaration of Helsinki.^29^ The present registry-based study was approved by the Danish Data Protection Agency. By Danish law, ethical approval and informed consent are not required for registry-based epidemiological studies. The study is reported according to the Strengthening the Reporting of Observational Studies in Epidemiology (STROBE) reporting guideline.^30^

Denmark has a genetically homogenous population of 5.8 million inhabitants (in 2019) who are all registered in the Danish Civil Registration system, in which they are recorded with a unique 10-digit personal identification number that allows follow-up in national health and administrative registries. The Danish National Patient Registry (DNPR), established in 1977, contains records of all patient-level discharges from private and public hospitals, and since 1995, it has included all treatments in hospital-based outpatient clinics. Reporting is compulsory and linked to the allocation of resources. At least 1 diagnostic code from the *International Statistical Classification of Diseases and Related Health Problems, Tenth Revision *(*ICD-10*) is recorded for each patient-hospital contact. Because health care is free in Denmark, complete case ascertainment is expected in these high-quality, validated registries, implying minimal bias.^31^

In Denmark, children suspected of PP are evaluated in 1 of 18 pediatric departments according to national guidelines. We identified all hospital patient records of first-time diagnoses for boys and girls for our main area of interest, CPP (*ICD*-*10* codes E22.8 and E30.1) and normal variant puberty, including PT (*ICD*-*10* code E30.8) and PA (*ICD*-*10* code E27.0), in the DNPR from 1998 to 2017 to measure changes in incidence during this time period. The generally accepted maximum diagnostic age limit for PP remains 8 years for girls and 9 years for boys and has not changed since the 1950s. Hence, the present diagnostic criteria for PP include onset of puberty before age 8 years for girls and age 9 year for boys. However, there is an established 6-month to 12-month lag time between the first observation of signs of puberty reported by parents and the establishment of a record (ie, diagnosis by a pediatrician) in the DNPR. This is explained by the time taken for families to notice and seek medical attention from their general practitioner, for the general practitioner to make a pediatric referral, and for the specialist appointment to occur. The clinical and biochemical evaluation of each child is electronically recorded in the patient record file and automatically transferred to DNPR (ie, as final diagnosis). This lag has previously been reported in a Belgian study^32^ and is in accordance with our previous experience, in which we reported a mean lag time of 7 months (range, 0-66 months) in girls and 4 months (range, 0-12 months) in boys.^22^ Thus, to account for this lag in our main analyses, we included first-time diagnoses if they were recorded in the DNPR between age 0 to 9 years for girls and 0 to 10 years for boys. In sensitivity analyses and to address potential overestimation of annual incidences due to the introduction of a 12-month lag for diagnosis, we also assessed trends only considering first-time diagnoses if they were recorded in the DNPR between the ages of 0 to 8 years for girls and 0 to 9 years for boys. The age of diagnosis was defined on the date of the respective incident diagnosis.

To determine the potential association of genetics and/or early life environmental exposures of children emigrating to Denmark with the outcomes studied, we stratified our findings into 3 groups according to the definitions of ethnic group adapted directly from Statistics Denmark. Statistics Denmark is a Danish governmental organization under the Ministry for Economic and Interior Affairs responsible for creating statistics on Danish society, including immigration, employment, demographic characteristics, and trade balance. Children with Danish origin were defined as having at least 1 parent who was both a Danish citizen and born in Denmark. Children born in Denmark who had 2 parents who were born abroad and not Danish citizens were defined as second-generation immigrants, and children not born in Denmark who had 2 parents who were born abroad and not Danish citizens were defined as first-generation immigrants. All data relating to immigration status, place of birth, and information on the total number of children (age and sex) within the relevant age groups at risk living in Denmark on January 1 each year within the study period were obtained from Statistics Denmark.^33^ Immigrant families, who might only be in Denmark for a short period while their application for asylum is considered, are not recorded in population registers. Consequently, these data were excluded.

### Statistical Analysis

First, the crude distribution of incident cases according to sex, immigration status, and diagnostic subgroup was recorded. The mean incidences of each disorder estimated by computing an annual mean of the total number of children in the reported age group in Denmark during the entire 20-year period and in 5-year subperiods by sex and immigration group, within the entire study period from 1998 to 2017.

Second, the trend in yearly incidence rates were estimated for our primary outcome, CPP, then for benign forms of PP, namely PT and PA, separately and for the sum of all diagnosis subgroups. Data are visualized as a function of calendar year.

Finally, age-specific mean incidence rates of true CPP, PT, and PA per 10 000 children were calculated. SAS version 9.4 (SAS Institute Inc) was used to calculate by standard methods. No prespecified level of statistical significance was set.

## Results

A total of 8596 children (7770 [90.4%] girls; median [interquartile] age at diagnosis for boys, 8.0 [7.1-9.0] years; for girls, 8.0 [7.8-8.5] years) were registered with a first-time diagnosis of CPP, PT, or PA during the 20-year study period, corresponding to 430 new cases per year in Denmark. Most children (7391 [86.0%]) diagnosed with these disorders had Danish origin (6671 [90.3%] girls), corresponding to 370 new cases in children with Danish origin per year (Table 2). The mean (SD) 20-year annual population of girls and boys in Denmark at risk were 321 702 (15 795) and 372 154 (17 055), respectively, of whom more than 89% had Danish origin (girls, 288 909 [12 334]; boys 334 378 [12 789]) (Table 2).

**Table 2.** Total Number of Incident Cases and Mean Annual 20-Year Incidence of Central Precocious Puberty, Premature Thelarche, Premature Adrenarche, and All Diagnoses, by Sex and Immigration Group, 1998 to 2017^a^

Immigration group	Annual population, mean (SD)^b^	Central precocious puberty			Premature thelarche			Premature adrenarche			Sum (all diagnoses)		
		Total incident cases, No.	Yearly cases, mean SD, No.^c^	Incidence (95% CI) per 10 000 per year	Total incident case, s, No.	Yearly cases, mean (SD), No.^c^	Incidence (95% CI) per 10 000 per year	Total incident cases, No.	Yearly cases, mean (SD), No.^c^	Incidence (95% CI) per 10 000 per year	Total incident cases, No.	Yearly cases, mean (SD), No.^c^	Incidence (95% CI) per 10 000 per year
**Girls**													
Danish origin^d^	288 909 (12 334)	5288	264.4 (123.4)	9.2 (8.0 to 10.3)	637	31.7 (23.6)	1.1 (0.7 to 1.5)	750	37.5 (33.1)	1.3 (0.9 to 1.7)	6671	333.6 (171.9)	11.5 (10.3 to 12.8)
First-generation immigrant^e^	26 918 (17 70)	740	37.0 (17.7)	13.7 (9.3 to 18.2)	58	2.9 (3.4)	1.1 (−0.1 to 2.3)	106	5.3 (4.4)	2.0 (0.3 to 3.6)	904	45.2 (23.4)	16.8 (11.9 to 21.7)
Second-generation immigrant^f^	5875 (1691)	167	8.4 (4.7)	14.2 (4.6 to 23.9)	10	0.5 (0.7)	0.9 (−1.5 to 3.2)	18	0.9 (1.4)	1.5 (−1.6 to 4.7)	195	9.8 (5.8)	16.6 (6.2 to 27.0)
**Boys**													
Danish origin^d^	334 378 (12 789)	583	29.2 (15.1)	0.9 (0.6 to 1.2)	NA	NA	NA	137	6.9 (5.4)	0.2 (0.1 to 0.4)	720	36.0 (19.0)	1.1 (0.7 to 1.4)
First-generation immigrant^e^	30 510 (2297)	64	3.2 (2.5)	1.0 (−0.1 to 2.2)	NA	NA	NA	25	1.3 (1.1)	0.4 (−0.3 to 1.1)	89	4.5 (3.1)	1.5 (0.1 to 2.8)
Second-generation immigrant^f^	7266 (1969)	14	0.7 (1.0)	1.0 (−1.3 to 3.2)	NA	NA	NA	3	0.2 (0.4)	0.2 (−0.8 to 1.3)	17	0.9 (1.0)	1.2 (−1.3 to 3.7)

Abbreviation: NA, not applicable.

^a^ The generally accepted maximum diagnostic age limit for precocious puberty remains 8 years for girls and 9 years for boys. There is an established 6-month to 12-month lag between the first observation of signs of puberty reported by parents and the establishment of a record (ie, diagnosis by a pediatrician), applied here.

^b^ Estimated by computing the annual mean of the total population of children in the reported age, sex, and immigration group in Denmark during the 20-year period.

^c^ Estimated by computing the annual mean of the number of incident cases by diagnosis, age group, sex, and immigration group in Denmark during the 20-year period.

^d^ Defined as a child with at least 1 parent who is both a Danish citizen and born in Denmark.

^e^ Defined as a child who was born in Denmark with neither parent who is both a Danish citizen and born in Denmark.

^f^ Defined as a child who was not born in Denmark with neither parent who is both a Danish citizen and born in Denmark. If no information was available on the child’s parents, immigration status was based on the child’s place of birth, otherwise it was based on the mother’s place of birth.

The 20-year mean annual incidence of CPP, PT, PA, and the sum of these diagnoses per 10 000 girls with Danish origin was 9.2 (95% CI, 8.0-10.3), 1.1 (95% CI, 0.7-1.5), 1.3 (95% CI, 0.9-1.7), and 11.5 (95% CI, 10.3-12.8), respectively (Table 2). For Danish boys, the 20-year mean incidence rates per 10 000 boys were 0.9 (95% CI, 0.6-1.2), 0.2 (95% CI, 0.1-0.4), and 1.1 (95% CI, 0.7-1.4) for CPP, PA, and the sum of the diagnoses, respectively (Table 2).

**Figure 1.**
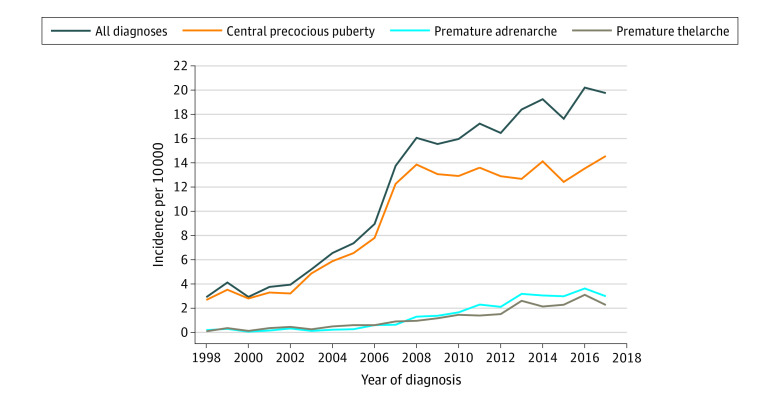
Trends in the Annual Incidence Among Girls With Danish Origin by Year of Incident Diagnosis, 1998 to 2017

Figure 1 shows the incidence in children with Danish origin, subdivided by year of diagnosis. Among girls, there was a 6-fold increase in the yearly incidence of CPP, from 2.6 per 10 000 to 14.6 per 10 000, during the 20-year period from 1998 to 2017, while we observed a 33-fold and 18-fold increase in the annual incidence of PT and PA, respectively, during the same time period (PT: 1998 incidence rate, 0.07 per 10 000 girls; 2017 incidence rate, 2.24 per 10 000 girls; PA: 1998 incidence rate, 0.16 per 10 000 girls; 2017 incidence rate, 2.96 per 10 000 girls) (Figure 1). In sensitivity analyses without the application of the 12-month lag for diagnosis, similar trends were detected, with a 6-fold increase in the yearly incidence of CPP from 2.1 per 10 000 girls to 12.9 per 10 000 during the 20-year period in girls with Danish origin, while for the other disorders we observed a 31-fold and 16-fold increase in the annual incidence of PT and PA, respectively, during the same time period (Table 1; eFigure in the Supplement). For boys with Danish origin, the annual incidence trend demonstrated a 15-fold increase in diagnosis of CPP, from 0.1 per 10 000 boys to 2.1 per 10 000 boys during this 20-year period, and in 2017, the incidence of CPP was 9-fold higher among girls with Danish origins compared with boys with Danish origin (data not shown).

**Figure 2.**
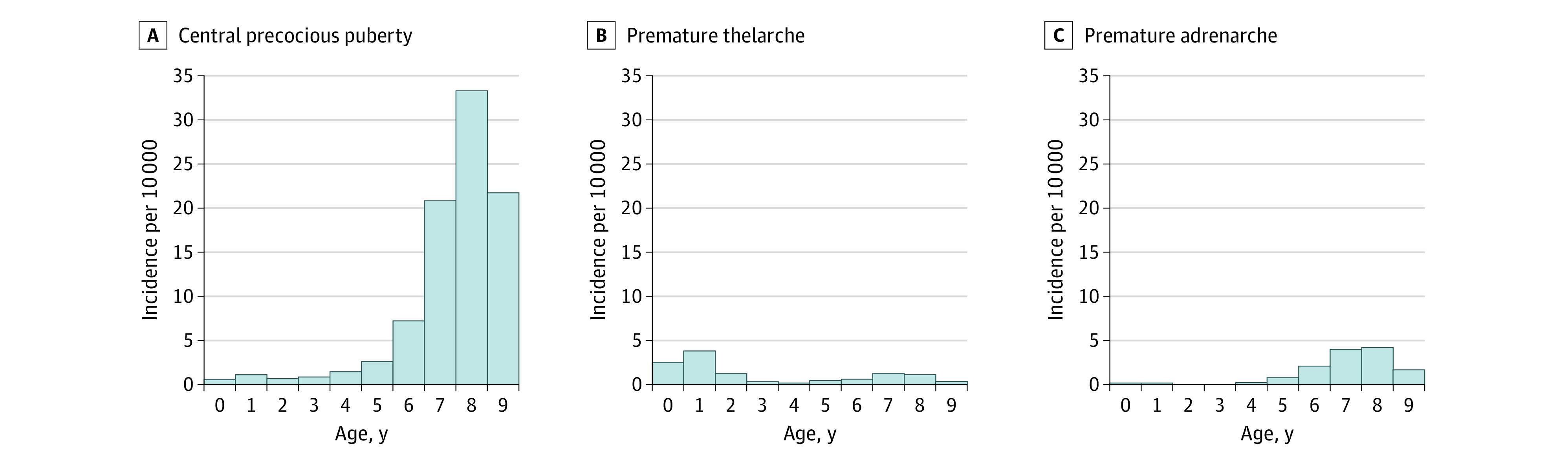
Age-Specific Mean Incidence Among Girls With Danish Origin, 1998 to 2017

The trends in age-specific incidence of CPP, PT, and PA among girls with Danish origin are shown in Figure 2. The incidence of CPP was less than 5 per 10 000 girls with Danish origin aged 5 years or younger and is highest at age 8 years, with an age-specific incidence of 34 per 10 000 (Figure 2). For PA, age-specific incidence is highest at ages 7 to 8 years, while for PT, age-specific incidence is highest between infancy and 2 years (Figure 2). For boys with Danish origin, the incidence of CPP was much lower (ie, <1 per 10 000) and increased to 2 to 3 per 10 000 in boys older than 8 years (data not shown).

Table 2 also shows the mean annual incidence of CPP, PT, and PA according to first-generation and second-generation immigrant groups during this 20-year period. The 20-year mean incidence of CPP and PA among girls in the first-generation and second-generation immigrant groups were greater than that of girls with Danish origin. The incidence rate for CPP per 10 000 girls in the first-generation and second-generation groups were 13.7 (95% CI, 9.3 to 18.2) and 14.2 (95% CI, 4.6 to 23.9), respectively, the incidence rate for PA per 10 000 girls in the first-generation and second-generation groups were 2.0 (95% CI, 0.3 to 3.6) and 1.5 (95% CI, −1.6 to 4.7), respectively. The annual incidence of thelarche was similar among all girls (Table 2). No marked differences between immigrant groups were observed among boys (Table 2). When assessing the time trends in mean 5-year incidences of each disorder, a steady increase was observed for all children (boys and girls) with Danish origin (eTable in the Supplement).

## Discussion

In this 20-year nationwide registry-based cohort study, we detected a substantial upward trend in the annual incidence of CPP and normal variant puberty (ie, PT and PA) from 1998 to 2017 in girls with Danish origin. Annual incidences were several-fold lower for boys with Danish origin, but a similar substantial upward secular trend was detected. The annual incidence of CPP and PA was substantially greater in girls with non-Danish origins compared with girls with Danish origin.

While national data for CPP are sparse, 5 other studies (3 in Europe^22,24,25^ and 2 in Korea^26,27^) have reported the incidence of CPP (Table 1). In contrast to our previous study,^22^ in which no upward trend in the annual incidence of CPP was detected during a 9-year period from 1993 to 2001 in girls or boys,^22^ our new data during the 20-year period from 1998 to 2017 demonstrate a 3-fold and 2-fold increased incidence of CPP in girls and boys, respectively. These trends are deeply concerning for several reasons. First, there has been considerable debate about whether current criteria for the diagnosis of PP, defined as any sign of puberty in girls before age 8 years and boys before 9 years, are appropriate in contemporary populations. If indeed there has been a downward trend in the reference age of pubertal onset, this may have led to more girls falsely diagnosed with PP, reflecting outdated reference ranges. In addition to the potential adverse psychosocial implications of this diagnosis, this may also include unjustified hospitalization, brain magnetic resonance imaging (potentially under general anesthesia) to rule out central nervous system pathologies, and a treatment with GnRH agonists for several years that may not have been needed. Second, an increasing incidence of premature sexual development in younger girls potentially is associated with adverse implications for long-term health. Third, secular trends across populations in the onset of puberty without a clear indication of the underlying mechanism is concerning, given that there are no evidence-based preventive interventions available. The mechanisms underlying this increasing trend in incidence of CPP and its benign variants reported here are uncertain. Hereditary factors play a major role in the timing of puberty^34,35^ but cannot account for the substantial increase observed in our study because genetic susceptibility is considered constant. Childhood obesity is associated with earlier age at puberty and has increased in Denmark during this 20-year time period^36^; however, body mass index did not explain the earlier breast development in Denmark observed in a cohort of healthy girls in 2005 compared with 1992,^17^ and the contribution of obesity to national trends in PP remains uncertain.^1,37,38^ Other potential mechanisms, including prenatal and postnatal exposures to endocrine disruptors, early childhood nutrition, international adoption, and physical activity, may influence the endogenous endocrine milieu and potentially affect maturation and puberty timing.^1^ Our registry-based study does not have access to these individual data, so these mechanisms could not be explored.

The potential influence of environmental exposure to endocrine disruptors has been addressed in a 2018 French national study^25^ in which the incidence of CPP during a 3-year period from 2011 to 2013 was evaluated by geographical location. The authors reported that CPP was greater in children living in agricultural areas, suggesting that exposure to agricultural endocrine disruptors may be associated with CPP.^25^ That study used similar inclusion criteria and diagnostic criteria as our study, with comparable annual incidences for CPP.^25^

Two Korean studies^26,27^ based on national health insurance databases, which collect all hospital contacts, have corroborated the global upward secular trend in CPP. The first study reported an increased incidence of CPP from 0.33 per 10 000 girls to 5.04 per 10 000 girls and from 0.03 per 10 000 boys to 0.12 per 10 000 boys between 2004 and 2010 using a population of 12 351 children evaluated for PP.^26^ The subsequent Korean national study reported an increase from 8.94 per 10 000 girls to 41.53 per 10 000 girls and from 0.16 per 10 000 boys to 1.47 per 10 000 boys between 2008 and 2014.^26,27^ The mean incidence of CPP was several fold higher in these Korean studies compared with our data. Given that these the studies did not use the same inclusion criteria, direct comparisons can only be made with caution, but these differences may potentially indicate the importance of ethnicity in this condition.

A Spanish observational study of 34 pediatric endocrinology units (including 250 patients)^24^ estimated the annual incidence of CPP at 0.06 per 10 000 children in 2008 to 2009, substantially lower than other European studies. Unlike in Denmark, health care is not free in Spain, which may lead to a noncomplete case ascertainment and underestimation of cases in that study.

To our knowledge, this is the first study to report the national incidence of PA and PT. Our findings of an increased incidence of PT is consistent with a global reduction of 3 months per decade in the reference age at pubertal onset, as measured by thelarche, the first clinical sign of puberty. This was summarized in our 2020 systematic review and meta-analysis^28^ of 30 studies including more than 145 000 girls from 7 regions of the world (eg, the United States, Europe, Asia).^28^

Although we find the upward trends in incidence of CPP concerning, there has also been considerable debate regarding whether present guidelines of CPP are appropriate in contemporary populations. There may be basis for revision of reference ranges for pubertal onset based on new thorough clinical investigations of CPP diagnosis and interrelation with bone age advancement and hormonal changes in the general population.

Screening programs identifying early puberty could lead to preventative health measures given that early pubertal onset is consistently associated with adverse short-term and long-term health outcomes. If there is an international shift in the reference ranges for puberty in the general population, this carries implications for the current diagnostic criteria, which may need to be updated to reflect this change.

### Strengths and Limitations

In Denmark, individual-level information on disease has been collected in nationwide registries for several decades. Because health care is free, these high-quality registries have complete disease ascertainment with minimal bias in data collection owing to socioeconomic status. Denmark has a genetically homogenous population (>98% White individuals); thus, the results of the study would be generalizable to other similar populations.

We were unable to identify children who were adopted (approximately 0.8% of Danish children), so approximately 59 cases identified as having Danish origin could have been adopted and belonged to a different ethnic group. However, we do not expect this number to skew results substantially.

Our study was registry-based, and although Danish registries are known for their completeness and validity, there are still some limitations. First, none of the *ICD*-*10* codes were validated by investigation of hospital records, which may have affected the number of cases included. Thus, it is not known to what degree patients with registration of the *ICD*-*10* codes E22.8 and E30.1 met all diagnostic criteria for progressive CPP. Second, there is a chance that the initial diagnosis coding of a child suspected of having CPP was not updated appropriately when subsequent investigation did not confirm the diagnosis. However, in our previous epidemiological study,^22^ we performed a rigorous validation of registered diagnoses and showed that 96% of registered cases were classified correctly as a disorder of pubertal development including CPP, PA, and PT. In addition, any potential minor variations in registration of *ICD*-*10* codes would be random and would not affect the overall trends we observed.

During recent decades, there has been growing parental awareness of and concerns regarding earlier puberty due to media coverage in Denmark. This may have increased the number of children referred to our specialist service and other public and private Danish hospitals.

## Conclusions

This nationwide, registry-based cohort study during 20 years demonstrated a substantial increase not only in PT and PA but also in true CPP. These findings have implications for short-term and long-term health and potentially for the international reference classification of age at puberty.

## Notes

Supplement. **eTable. **Mean 5-Year Incidence of Central Precocious Puberty, Premature Adrenarche, and Premature Thelarche, by Sex and Immigration Group, 1998 to 2017 **eFigure. **Sensitivity Analyses, Comparison of Trends in the Annual Incidence Among Girls With Danish Origin by Year of Incident Diagnosis, 1998 to 2017
