# Supplementary material for: Trends in the Incidence of Central Precocious Puberty and Normal Variant Puberty Among Children in Denmark, 1998 to 2017
Source: JAMA Netw Open. 2020 Oct 12;3(10):e2015665. doi: 10.1001/jamanetworkopen.2020.15665 (PMC7550972; doi:10.1001/jamanetworkopen.2020.15665)
Supplement: Supplement. — eTable. Mean 5-Year Incidence of Central Precocious Puberty, Premature Adrenarche, and Premature Thelarche, by Sex and Immigration Group, 1998 to 2017 eFigure. Sensitivity Analyses, Comparison of Trends in the Annual Incidence Among Girls With Danish Origin by Year of Incident Diagnosis, 1998 to 2017 [file jamanetwopen-e2015665-s001.pdf]

Supplemental Online Content

Bräuner EV, Busch AS, Eckert-Lind C, Koch T, Hickey M, Juul A. Trends in the incidence of central precocious puberty and normal variant puberty among children in Denmark, 1998 to 2017. *JAMA Netw Open*. 2020;3(10):e2015665. doi:10.1001/jamanetworkopen.2020.15665

eTable. Mean 5-Year Incidence of Central Precocious Puberty, Premature Adrenarche, and Premature Thelarche, by Sex and Immigration Group, 1998 to 2017

eFigure. Sensitivity Analyses, Comparison of Trends in the Annual Incidence Among Girls With Danish Origin by Year of Incident Diagnosis, 1998 to 2017

This supplemental material has been provided by the authors to give readers additional information about their work.

© 2020 Bräuner EV et al. *JAMA Network Open.*

eTable. Mean 5-Year Incidence of Central Precocious Puberty, Premature Adrenarche, and Premature Thelarche, by Sex and Immigration Group, 1998 to 2017

Sex (age group)	Ethnic group	Period	Average yearly population[a]	Central Precocious Puberty		Premature Thelarche		Premature Adrenarche		Sum (all diagnoses)	
				Average yearly number of cases (N)[b]	Incidence per 10 000 per year	Average yearly number of cases (N)[b]	Incidence per 10 000 per year	Average yearly number of cases (N)[b]	Incidence per 10 000 per year	Average yearly number of cases (N)[b]	Incidence per 10 000 per year
Girls (0-9 years)	Group 1 (Danish Origin)[c]	1998 - 2002	301,936	93.0	3.08	7.6	0.25	5.4	0.18	106.0	3.51
		2003 - 2007	296,353	220.8	7.45	16.2	0.55	10.2	0.34	247.2	8.34
		2008 - 2012	286,841	380.2	13.25	36.4	1.27	49.4	1.72	466.0	16.2
		2013 - 2017	270,508	363.3	13.44	66.4	2.45	85	3.14	515.0	19.0
	Group 2 (Descendant)[d]	1998 - 2002	24,712	14.8	5.99	0.4	0.16	0.2	0.08	15.4	6.23
		2003 - 2007	27,739	30.8	11.10	1.8	0.65	2.4	0.87	35.0	12.61
		2008 - 2012	27,008	52.4	19.40	2.4	0.89	8.2	3.04	63.0	23.33
		2013 - 2017	28,214	50.0	17.72	7.0	2.48	10.4	3.69	67.4	23.88
	Group 3 (Immigrant)[e]	1998 - 2002	5,808	5.0	8.61	0.4	0.69	0	0	5.4	9.30
		2003 - 2007	4,783	6.4	13.38	0.4	0.84	0	0	6.8	14.22
		2008 - 2012	4,744	7.6	16.02	0.2	0.42	1.0	2.11	8.8	18.55
		2013 - 2017	8,165	14.4	17.64	1.0	1.22	2.6	3.18	18.0	22.04
Boys (0-10 years)	Group 1 (Danish Origin)[c]	1998 - 2002	348,166	12.4	0.36	-	-	1.4	0.04	13.8	0.40
		2003 - 2007	341,727	18.6	0.54	-	-	4.4	0.13	23.0	0.67
		2008 - 2012	332,027	42.6	1.28	-	-	7.2	0.22	49.8	1.50
		2013 - 2017	315,593	43.0	1.36	-	-	14.4	0.46	57.4	1.82
	Group 2 (Descendant)[d]	1998 - 2002	27,308	1.2	0.44	-	-	0.2	0.07	1.4	0.51
		2003 - 2007	31,329	2.4	0.77	-	-	0.6	0.19	3.0	0.96
		2008 - 2012	30,967	5.8	1.87	-	-	1.4	0.45	7.2	2.33
		2013 - 2017	32,441	3.4	1.05	-	-	2.8	0.86	6.2	1.91
	Group 3 (Immigrant)[e]	1998 - 2002	7,470	0.6	0.80	-	-	0	0	0.6	0.80
		2003 - 2007	5,952	0.4	0.67	-	-	0	0	0.4	0.67
		2008 - 2012	5,834	0.2	0.34	-	-	0.2	0.34	0.4	0.69
		2013 - 2017	9,805	1.6	1.63	-	-	0.4	0.41	2.0	2.04

[a]Estimated by computing the annual mean of the population of children in the reported age group, sex and ethnic group in Denmark during the specified 5-yr periods.
[b]Estimated by computing the annual mean of the number of incident cases by diagnosis, age group, sex and ethnic group in Denmark during the specified 5-yr periods.
[c]At least one parent who is both a Danish citizen and born in Denmark (Danish origin).
[d]Born in Denmark, but neither parent is both a Danish citizen and born in Denmark (2nd generation immigrant).
[e]Not born in Denmark and neither parent is both a Danish citizen and born in Denmark. If no information is available on the child's parents, the land of origin is based on the child's place of birth, otherwise the land of origin is based on the mother's land of origin (1st generation immigrant)

© 2020 Bräuner EV et al. *JAMA Network Open.*

eFigure. Sensitivity Analyses, Comparison of Trends in the Annual Incidence Among Girls With Danish Origin by Year of Incident Diagnosis, 1998 to 2017

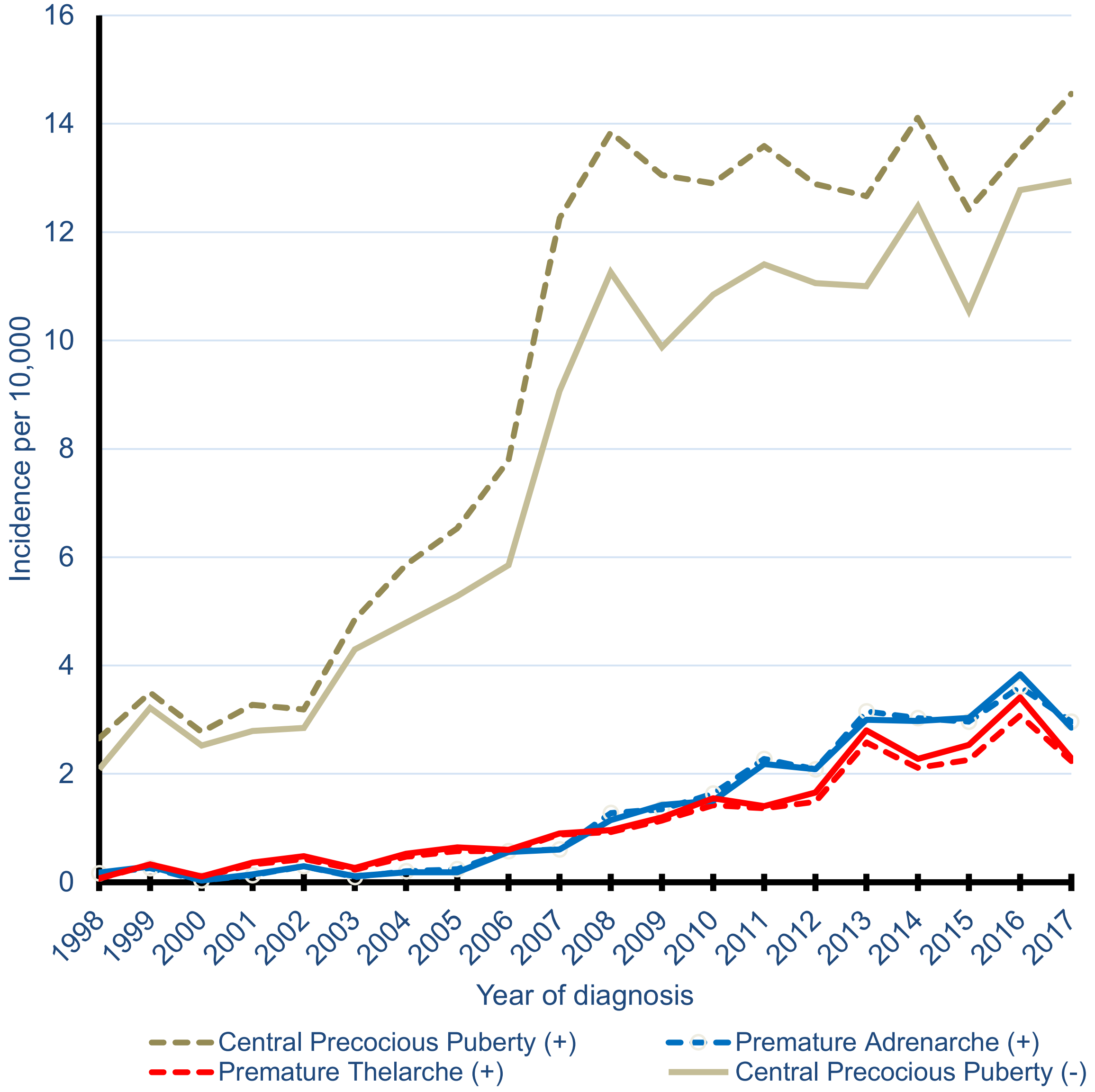

Broken lines indicate results with 1-year lag; sold line indicates results with no lag.

© 2020 Bräuner EV et al. *JAMA Network Open.*
